# A Quantitative Method to Track Protein Translocation between Intracellular Compartments in Real-Time in Live Cells Using Weighted Local Variance Image Analysis

**DOI:** 10.1371/journal.pone.0081988

**Published:** 2013-12-20

**Authors:** Guillaume Calmettes, James N. Weiss

**Affiliations:** UCLA Cardiovascular Research Laboratory, Departments of Medicine (Cardiology) and Physiology, David Geffen School of Medicine, University of California Los Angeles, Los Angeles, California, United States of America; University of California, Berkeley, United States of America

## Abstract

The genetic expression of cloned fluorescent proteins coupled to time-lapse fluorescence microscopy has opened the door to the direct visualization of a wide range of molecular interactions in living cells. In particular, the dynamic translocation of proteins can now be explored in real time at the single-cell level. Here we propose a reliable, easy-to-implement, quantitative image processing method to assess protein translocation in living cells based on the computation of spatial variance maps of time-lapse images. The method is first illustrated and validated on simulated images of a fluorescently-labeled protein translocating from mitochondria to cytoplasm, and then applied to experimental data obtained with fluorescently-labeled hexokinase 2 in different cell types imaged by regular or confocal microscopy. The method was found to be robust with respect to cell morphology changes and mitochondrial dynamics (fusion, fission, movement) during the time-lapse imaging. Its ease of implementation should facilitate its application to a broad spectrum of time-lapse imaging studies.

## Introduction

Proteins are often motile, shuttling between different subcellular destinations to perform various functions. Visualization and quantification of a protein's subcellular location and movement over time is therefore important for understanding how proteins function inside cells. Traditional approaches for monitoring protein translocation have relied on biochemical methods to measure protein levels in various cellular compartments over time (e.g. western blotting subcellular fractions at sequential time points). While these approaches have produced seminal advances in elucidating molecular mechanisms of protein function, they also have limitations. For example, they are not suitable for tracking protein translocation in single cells, limiting kinetic analysis to populations of cells. They also have limited time resolution due to tissue destruction required for biochemical assays at each time point. In addition, protein localization may be sensitive to the concentration of cytosolic ions and metabolites that are likely to be lost during the fractionation procedure [1].

The development of techniques to label proteins with genetically-encoded fluorescent tags, combined with advances in live cell fluorescent microscopy, has circumvented many of these limitations. In particular, these new techniques permit direct visualization of biochemical processes in living cells in real–time. A current challenge is to couple image processing techniques with statistical and computational tools to interpret and extract quantitative information from the vast amounts of unstructured image data generated by time-lapse imaging experiments.

Here we demonstrate a reliable and easy-to-implement quantitative image processing method to assess protein translocation between subcellular compartments in living cells, based on the computation of the spatial variance of time-lapse microscope images, which minimizes user-introduced biases. To demonstrate the usefulness and advantages, we first validated the method using simulated images and then applied the technique to analyze the translocation of fluorescently-labeled hexokinase (HK), a key glycolytic enzyme which shuttles between the cytoplasm and mitochondria. Currently, translocation of fluorescently-labeled proteins between intracellular compartments is most commonly quantified as ratio of fluorescence intensity between two user-defined intracellular regions of interest (ROI) in microscopy images. In the case of HK, an ROI with a high concentration of mitochondria is compared to an adjacent ROI with few mitochondria [2], [3]. While this method of measurement is generally useful, it suffers from three major drawbacks: (i) The choice of the ROI is arbitrary and subject to investigator bias (ii) Appropriate ROI can only be defined if the discrete organellar compartments are easily identifiable and separable in the images, as, for example, in Chinese Hamster Ovary (CHO) cells or neonatal cardiac myocytes in which mitochondria are concentrated in the perinuclear zone and sparse elsewhere. However, the method is problematic for cell types with a uniformly distributed organellar network, such as the mitochondrial network in adult cardiac myocytes. (iii) The ROI is also sensitive to changes in cell shape and migration of organelles throughout the cell during the time course of the experiment, making readjustment of the ROI necessary to avoid error. The spatial variance method described herein minimizes all of these shortcomings.

## Methods

### Ethics statement

This study was approved by the UCLA Chancellor's Animal Research Committee (ARC 2003-063-23B) and performed in accordance with the Guide for the Care and Use of Laboratory Animals published by the United States National Institutes of Health (NIH Publication No. 85-23, revised 1996) and with UCLA Policy 990 on the Use of Laboratory Animal Subjects in Research (revised 2010).

### Cell preparation

Animals were anesthetized with 2% isoflurane. Adequacy of anesthesia was assessed by monitoring the respiratory rate as well as the loss of response to toe pinch. Animals were then injected with sodium pentobarbital (100 mg/kg, i.p.) and hearts were rapidly removed to isolate ventricular myocytes. Neonatal rat ventricular myocytes (NRVM) were enzymatically isolated by standard methods [4]. Briefly, hearts harvested from 2- to 3-day-old neonatal Sprague-Dawley rats were digested with collagenase (0.02%; Worthington Biochemical Corp, Lakewood, NJ) and pancreatin (0.06%; Sigma-Aldrich, St. Louis, MO). Myocytes were isolated with the use of a Percoll (Pharmacia Biotech AB, Uppsala, Sweden) gradient and plated on 35 mm glass bottom culture dishes. Adult rat ventricular myocytes (ARVM) were enzymatically isolated from the hearts of 3-to 4-month old male Fisher rats as described previously [5]. Briefly, following anesthesia, hearts were removed and perfused retrogradely at 37°C in Langendorff fashion with nominally Ca^2+^-free Tyrode's buffer containing 1.2 mg/ml collagenase type II (catalog number 4176; Worthington) and 0.12 mg/ml protease type XIV (catalog number P5147; Sigma) for 25–28 min. After washing out the enzyme solution, hearts were subsequently removed from the perfusion apparatus and gently agitated to dissociate the myocytes. The Ca^2+^ concentration was gradually increased to 1.8 mmol/L over 30 min. This procedure typically yielded 40–60% of rod-shaped, Ca^2+^-tolerant myocytes that were then plated on 35 mm glass bottom culture dishes. NRVM and ARVM were cultured in DMEM high (25 mM) glucose medium supplemented with 6% (v/v) fetal bovine serum (FBS), penicillin (100 units/ml), streptomycin (100 units/ml) and 2 mM glutamine.

### Gene expression

Rat HKI and HKII were generously provided by Dr. J. Wilson. Fusion of rat HKs to YFP was accomplished by inserting a Bam H1 site at the last amino acid of the coding sequence and subcloning into a modified pEYFPN-1vector (Clontech). The modified pEYFPN-1 carried the mutations Q86K and A206K. All constructs were subcloned into the mammalian expression vectors utilizing the CMV promoter.

Overexpression of HKI and HKII in NRVM and ARVM was achieved with engineered adenoviruses encoding the constructs. Expression of the constructs was sufficiently high after 36–48 h (NRVM) and 72–96 h (ARVM) to perform microscopy imaging.

### Standard and confocal microscopy imaging

Standard microscopy images were acquired using an Olympus IX70 inverted microscope (Olympus, America Inc.) fitted with an Olympus plan apo 60×, 1.4 N.A. oil immersion objective and a cooled CCD camera (Model Quantix, Photometrics, Tucson, AZ). Imaging Workbench software was used for data acquisition. YFP (XF104-2) filter cube was purchased from Omega Optical Inc. Confocal images were acquired using on a Zeiss Axiovert 100 LSM inverted microscope fitted with a 60× water immersion objective (Zeiss C-Apochromat 63/1.2 W Corr). Zeiss Pascal 5 image software (Carl Zeiss, Inc., Thornwood, NY) was used for data acquisition.

### Solutions and experimental techniques

The bath solution for cell imaging consisted of (in mM) 140 NaCl, 5 KCl, 1.1 MgCl2, 2.5 CaCl_2_, 10 HEPES, Glucose, 10, with the pH adjusted to 7.2 with KOH. For the anoxia/reoxygenation experiments, the cells were perfused with the same solution containing 5 mM of sodium dithionite (an oxygen-depleting agent, [6]–[8]) for 15 min followed by 10 min superfusion with the original solution. Solutions were perfused directly over the cells using a gravity fed eight ways perfusion device (Warner Instruments, Hamden, CT) with electrically controlled solenoids (The Lee Company, Westbrook, CT). Input and output of solution volumes to the recording chamber (35 mm glass bottomed culture dish) were equilibrated to maintain constant flow rates and pressures within the recording chamber. Experiments were carried out at room temperature (25°C).

### Numeric treatment and algorithm generation

Image analysis, algorithm generation, statistical analysis and simulations were implemented in the Python programming language [9], using the Numpy [10] and Scipy [11] packages that provide support for array manipulation and general scientific computation respectively [12].

### Statistical analysis

QQ-plot and Shapiro-Wilk tests were performed to assess the normality of the samples under analysis. The conventional percentile bootstrap-resampling approach with 10000 replications was used for estimating 95% CI as well as examining the significant difference between groups (effect size statistics) [13], [14]. All analyses were performed by subroutines for bootstrapping developed in the Python programming language, using the Numpy and Scipy packages, based on the code we previously published [13]. Effects were also analyzed with Mann-Whitney U tests. A *P* value<0.05 was considered statistically significant.

### Supplementary formulas

Equation of a two-dimensional Gaussian function
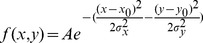
with A, the amplitude of the Gaussian; x_0_ and y_0_, the center of the Guassian in the plane (x, y); and σ_x_ and σ_y_, the spreads of the Gaussian in the x and y directions.
Volume under a 2D Gaussian: 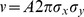

### Method overview

**Figure 1:**
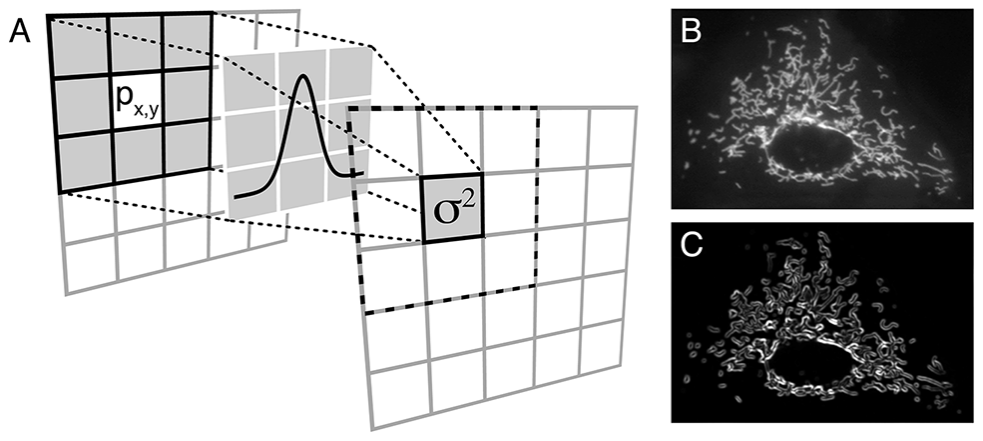
Gaussian weighted local variance computation process. **A**) During the process, the source pixel is replaced with a Gaussian weighted variance of all the pixels inside the chosen window size. Each pixel integer value in the source image is multiplied by the corresponding value in the overlying Gaussian kernel, and the variance of all the resulting products is computed. The gray value of the source pixel (P_x,y_) is then replaced by this Gaussian weighted local variance. This operation is repeated for each pixel in the original image. **B**) Source image and **C**) corresponding output image after application of the algorithm.

In cells, mitochondria form a network of discrete organelles, observable in microscopy images in the form of globules and/or filaments when labeled using mitochondria-specific dyes such as TMRM, or expressing fluorescently-tagged proteins which localize to mitochondria (figure 1B). In the latter case, if the protein molecules translocate from mitochondria to cytoplasm, a concomitant decrease of the mitochondrial fluorescence and increase of fluorescence inside the cytoplasm will be observed, resulting in the dilution of the mitochondrial fluorescence and making the interior of the cell more homogeneously fluorescent in the images. Therefore, in addition to the commonly used ratio-based measurement tracking the fluorescence intensity changes over time between an ROI containing a high concentration of mitochondria, and an ROI representing the adjacent cytosolic area with spare mitochondria [2], [3], assessment of fluorescent protein translocation can be also handled as a features detection problem, i.e. assessing the disappearance of the mitochondrial objects.

Image derivative based approaches such as the Sobel [15] and Canny [16] operators are among the most popular image processing algorithms using edge detection for object detection [17]. However, spatial variance mapping-based approaches [18]–[20] have been shown to be superior for object detection under conditions of blurred or low contrast images [18], [20]. By comparing the variation in pixel intensity from region to region throughout the entire cell, spatial variance also provides a quantitative measure of the overall spatial complexity inside a cell. Since mitochondria are discrete organelles, the spatial variance of grey scale values in a given window is large when fluorescence from a protein arises predominantly from mitochondria, and decreases as it is released and diffuses evenly throughout the cytoplasm.

In the spatial variance algorithm, the variance operator is a local neighborhood operation that calculates the sum of squares of the brightness differences from the mean for the neighborhood surrounding each pixel in the original image [17]. The variance value is small in regions of the image with uniform brightness and becomes large whenever sharp dark-bright transitions occur, which allow easy detection of organelles such as mitochondria (figure 1B and C). In our algorithm, for each pixel P_x,y_ in the input image (figure 1A), the variance was calculated for a given window size using the formula below which allows local computations to be performed simultaneously and efficiently within windows of many sizes [21]:
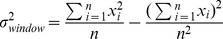
where x and n are the intensity of each pixel and the number of pixels within the window, respectively.

In practice, most windows are squared, providing uniform weight to all points within the window area, and zero weight to points outside this area. Computations performed within a squared window, however, may be overly sensitive to image points near the edge of the window, so that estimates of image properties change abruptly as the window is moved across pixels that represent image noise. This effect may be avoided by measuring properties within Gaussian-like windows in which the greatest weight is given to the pixels near the sample position and progressively less weight is given to more distant pixels [21]. The Gaussian outputs a ‘weighted average’ of each pixel's neighborhood, with the average weighted more towards the value of the central pixels. This is in contrast to the mean filter's uniformly weighted average. Because of this, a Gaussian filter provides gentler smoothing and preserves edges better than a similarly sized mean filter (this is illustrated in the figure S1).

Therefore, in our algorithm, the Gaussian-weighted variance (σ^2^) of all the pixels in a chosen window is computed, and the value is attributed to the central pixel (P_x,y_) of this window (figure 1A). The window is then moved by one pixel, and the operation is repeated, and so forth for all the pixels in the input image to obtain the variance map of the original image (figure 1B).

## Results

### Method validation

#### Simulated images of protein translocation

**Figure 2:**
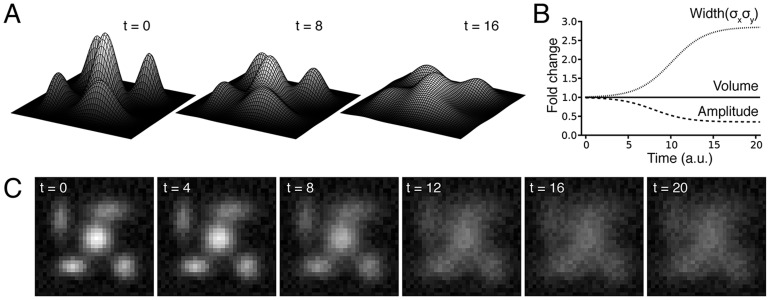
Simulation of hexokinase (HK) translocation from mitochondria to cytoplasm. **A**) Area of high fluorescence arising from mitochondrial HK-bound was simulated by a series of 2D Gaussians, whose redistribution of the fluorescence simulates the translocation from mitochondria to cytoplasm. **B**) During this process, the height and the width of each 2D Gaussian are changed so the volume under the 2D Gaussian is kept constant, to simulate a redistribution of the fluorescence over the entire cell. **C**) Top view of the simulation of the translocation of HK from mitochondria to cytoplasm. A uniform random noise is added to generate the final simulation images.

To test our method, we first generated artificial images simulating translocation of a protein from mitochondria to cytoplasm over time (figure 2). Mitochondrial protein-bound areas were modeled as isotropic or anisotropic 2D Gaussians with added random noise, and translocation was simulated through redistribution of the volume under the Gaussians over the adjacent area in the image (decrease in amplitude and increase in width, so that the volume is kept constant) (figure 2). In our experimental images, the mitochondria are approximately 5 pixels wide; therefore, in our computational images (30×30 pixels), we modeled mitochondria with 3 to 7 pixel-wide 2D Gaussians (figure 2).

**Figure 3:**
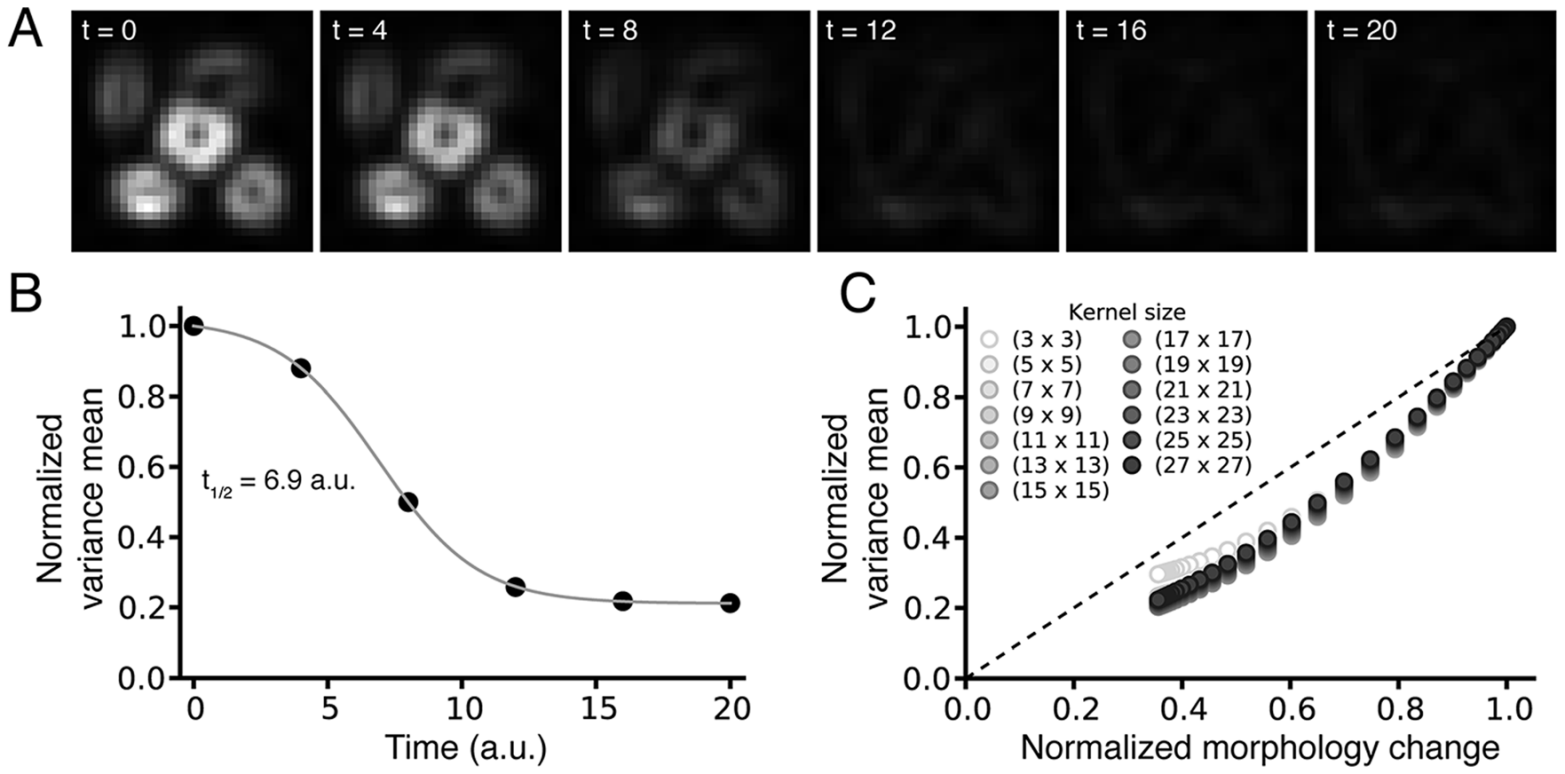
The local variance algorithm efficiently detects redistribution between compartments. **A**) Application of the weighted variance map algorithm to the model illustrated in figure 2. As seen in the images, the spatial local variance is high in the regions of high fluorescence and decreases over time as the fluorescence is redistributed over the whole image. **B**) From the time course of the change in variance, the time constant of dissociation can be measured. Note that this time constant (6.93 a.u.) is very close to the time constant used to model the translocation (7 a.u., figure 2). **C**) The plot of the local variance mean obtained by the analysis against the true degree of simulated protein translocation, which reveals that the analysis tends to be more sensitive to small changes in variance when the variance is high, whatever the kernel size used for the analysis. This property is an advantage to detect early translocation.

Computation of the spatial variance for the sequence of images in figure 2C simulating protein dissociation from mitochondria is presented in figure 3A. In our algorithm, the variance map command computes a map of the input image, where the intensity of a pixel in the output map represents the variance within the pixels window centered at the corresponding pixel of the input image. This operation is applied to all the pixels of the original image through a local neighborhood operation. Since mitochondria are discrete organelles, the spatial variance is large when fluorescence arises predominantly from mitochondria (figure 3A, first image). As the protein is released and diffuses evenly through the cytoplasm, intensity of the variance image decreases and objects become less and less detectable in the images (figure 3A, subsequent images). Calculation of the mean of the variance values for each image allows to quantify the decrease in variance signal as protein translocates to the cytoplasm over time (figure 3B). Dissociation time constant can be then extracted from the data (figure 3B).

To compensate for artifacts arising from bleaching of the preparation, the variance obtained for each image is normalized to the total amount of fluorescence in the image (figure S2). This is justified because we are interested in measuring the relative redistribution of a fluorophore between different cellular compartments, rather than the absolute changes in fluorescence variance which include bleaching as well as redistribution.

Note that our method is more sensitive than the region-of-interest (ROI) ratiometric method (ratio of fluorescence intensity between two intracellular ROI, usually an ROI on cellular area with high concentration of mitochondria, and an ROI representing the adjacent cytosolic area (Carrington et al., 1995)) (see figure S3A and S3B).

As shown in figure 3C, plotting the values obtained by the analysis against the function used to model the protein translocation reveals that our analysis tends to be more sensitive to small changes in variance when the variance is high. This property is an advantage to detect early translocation of the protein from mitochondria. Moreover, the window size used to compute the spatial variance of the images does not strongly affect the output of the analysis, as shown by the similar results obtained using windows from size 3-by-3 to 27-by-27.

#### Influence of mitochondrial motility

**Figure 4:**
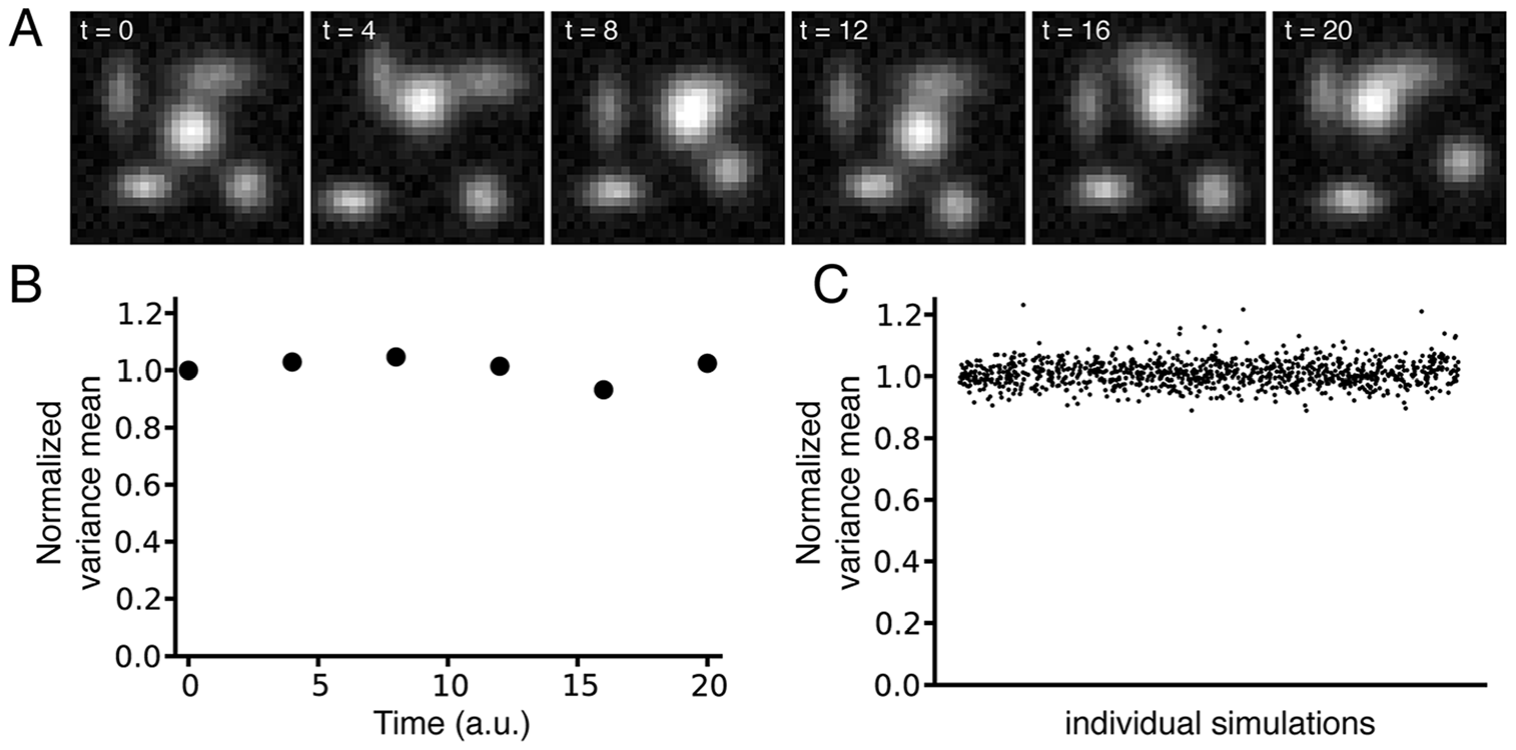
Robustness of the method when simulated mitochondria change position over time without otherwise redistributing their fluorescence. **A**) Example of a sequence of simulated images recapitulating mitochondrial motility by random assignment of the location of the 2D Gaussians, while the size and width of the 2D Gaussians are kept unchanged over time (mitochondrial motility but no protein translocation). **B**) The corresponding computed spatial local variance for the images remains almost constant. **C**) The robustness of the method was tested on images simulating 1,000 different patterns of mitochondrial motility over time. Overall, the spatial local variance measured for all the simulations remains constant.

We then tested the robustness of our analysis to mitochondrial motility, phenomenon commonly observed in time-lapse microscope imaging [22]. In this aim, we generated simulated images in which the location of the 2D Gaussians has been randomly assigned, without simulating protein translocation (size and width of the 2D Gaussians unchanged over time). An example of sequence of images recapitulating the movement of mitochondria over time that could be observed in experimental images is presented in figure 4A. Unlike the ratiometric method, which performs poorly when the objects in the images are motile (see figure S3C), our algorithm is insensitive to mitochondrial movement and a similar variance is calculated over time for this sequence of images (figure 4B). Figure 4C, in which the similar analysis has been extended to 1000 simulations with different degrees of mitochondrial motility, shows that our method is robust toward mitochondrial motility as similar spatial variance is observed for all the images.

To compare a Gaussian kernel over a unity kernel to compute the variance in our method, we did a statistical pair-wise comparison using the Bland-Altman framework [23] of the results obtained with the two kernels for different sequences of images simulating protein dissociation from mitochondria, without or with mitochondrial motility. As seen in the figure S4A, when the data obtained from the analysis of 1000 sequences of images simulating protein dissociation from nonmotile mitochondria using the Gaussian kernel are plotted against the data obtained with a unity kernel, all the points lie on the equality line. Moreover, the graphical depiction of differences between paired observations from the two methods versus their average (figure S4B) shows that, even if the unity kernel tends to give slightly higher values, the calculated mean difference between measurements (orange square in the figure) is not significantly different from zero, and the slope of the linear fit of the differences is horizontal. These results suggest that there is a very high degree of agreement between the two methods when only protein dissociation is modeled in the images. However, when the same analysis is done on sequences of images simulating dissociation of protein from motile mitochondria, the plot of the pair of measurements obtained from the two methods deviate from the equality line (figure S5A). Moreover, both the average in differences, indicator of the constant bias, and the slope of the regression of differences on means, which is a satisfactory method for detecting proportional bias [24], are statistically different from zero (figure S5). This over-estimation of the variance values when using the unity kernel compared to the Gaussian kernel to analyze images simulating protein translocation coupled to mitochondrial motility might be the consequence of the excessive sensitivity of the unity kernel to image points near the edge of the unity window giving very high values when the simulated moving mitochondria suddenly enter the window.

### Application to experimental data

**Figure 5:**
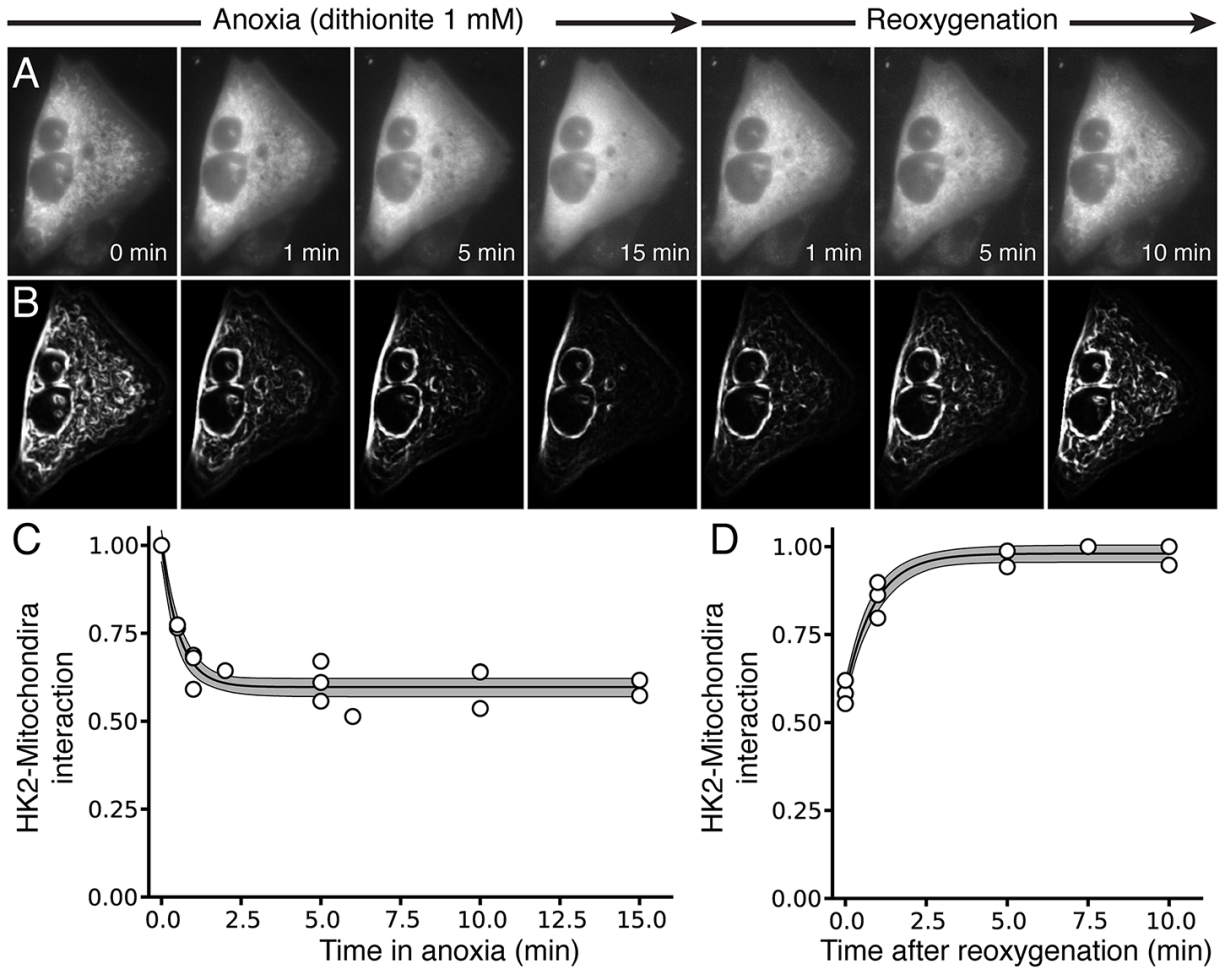
Hexokinase 2 translocation in living cells exposed to anoxia/reoxygenation. **A**) Snapshot of fluorescence in CHO cells were subjected to 15 min chemical anoxia using the oxygen scavenger dithionite (1 mM), followed by 10 min of reoxygenation. **B**) Corresponding spatial variance images. **C & D**) Plot of the average spatial variance at the various time points, showing the time course of the dissociation (C) and re-association (D) of HK2 with mitochondria during the anoxia/reoxygenation episode. Note that the measurement time points on differents cells varied, explaining the different number of measurements for each time point.

We validated our method on images obtained from CHO cells, NVRM and ARVM expressing hexokinase 2 (HK2) linked to YFP [25]. CHO cells were subjected to anoxia/reoxygenation to induce HK2 dissociation/reassociation from mitochondria (figure 5). The anoxia/reoxygenation episode was mimicked by exposing the cells for 15 min to a solution containing the oxygen scavenger dithionite (1 mM), followed by 10 min superfusion with the control solution (figure 5). At the beginning of the experiment, a large fraction of HK2 was bound to mitochondria, and upon exposure to anoxia, HK2 rapidly translocated to the cytosol (figure 5A). This effect was reversible as HK2 re-associated with mitochondria after reoxygenation (figure 5A). Application of our spatial variance analysis algorithm to the images (figure 5B) allowed us to quantify HK2 dissociation (figure 5C) and re-association (figure 5D) with mitochondria during the anoxia/reoxygenation episode, data from which useful information can be obtained, such as the half-time constant of dissociation/re-association.

**Figure 6:**
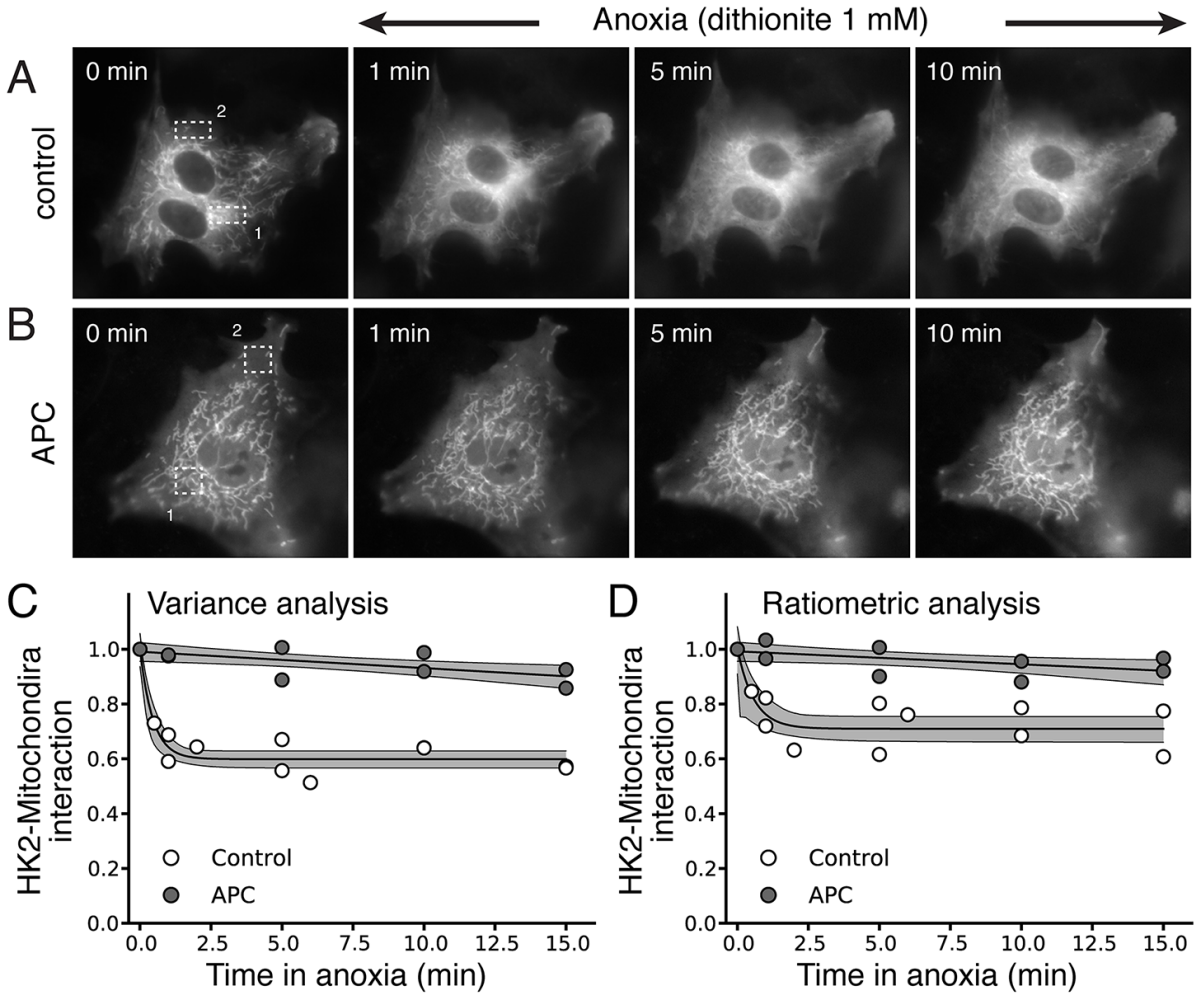
Anoxic preconditioning (APC) prevents HKII dissociation from mitochondria during anoxia in NRVM. **A & B**) Snapshots of fluoresence in NRVM during anoxia, illustrating rapid translocation of HKII-YFP from mitochondria to cytosol (A), which is prevented if NVRM are exposed to 3 short anoxic preconditioning exposures (1 min each) before the prolonged anoxia episode (B). **C & D**) Time course of changes in spatial variance (C) versus the ratiometric ROI method (D). For the ratiometric ROI methods, a ROI with a high concentration of mitochondria (1) was compared to a ROI placed into a region with few mitochondria (2) as illustrated in the series of images in A & B. Note that the measurement time points on differents cells varied, explaining the different number of measurements for each time point.

Similar findings were obtained in NRVM (Fig. 6C, control trace). In this case, the ROI method also performed reasonably well (Fig. 6D, control trace) even if the sensitivity of dissociation detection was lower (0.401, 95% CIs [0.37, 0.43], for the variance method versus 0.29, 95% CIs [0.25, 0.34], for the ratiometric method, p<0.05) (ROI #1 and #2 noted in the images were used respectively as area of high mitochondrial density and cytoplasmic area to perform the ratiometric method). To show that the measured dissociation was not an artifact of the method, we repeated the experiments of HK2 dissociation in NRVM after exposing the cells to anoxic preconditioning, which prevents HK2 dissociation from mitochondria [26]. In this case, no HK2 dissociation was observed in the images (Fig. 6B) or detected by our image analysis method (Fig. 6C, APC trace) and the ROI ratiometric method (Fig. 6D, APC trace).

**Figure 7:**
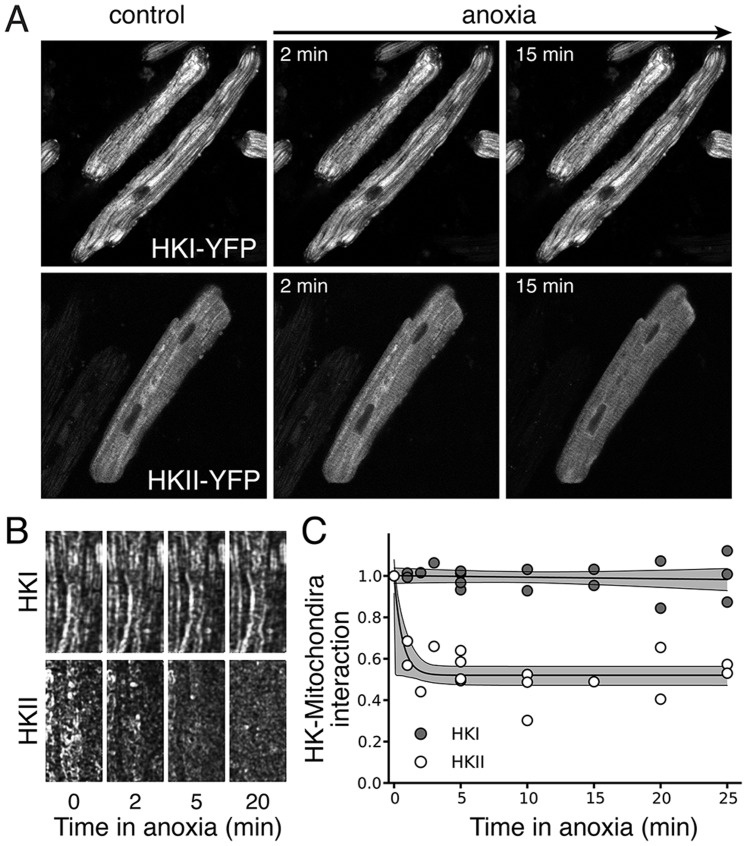
A) Fluorescence snapshots of AVRM expressing HKI-YFP (upper) or HKII-YFP under control conditions and after different periods of anoxia. Note that the mitochondrial network is very dense and uniformly dispersed, making difficult to define appropriate ROI if we wanted to apply the ratiometric ROI method. B) Corresponding spatial variance images. C) Average changes in spatial variance during anoxia, confirming that HK2, but not HK1, translocates to the cytoplasm during anoxia. Note that the measurement time points on differents cells varied, explaining the different number of measurements for each time point.

AVRM, on the other hand, represent a more challenging setting, since the dense uniformly dispersed mitochondrial network makes defining appropriate ROI complicated, requiring a small spatial scale which becomes sensitive to changes in cell position, cell morphology and mitochondrial migration. HK2-YFP was overexpressed in ARVM and the cells were submitted to anoxia (Fig. 7A, lower series of images). In those experiments, the spatial variance method (Fig. 7B) clearly identified translocation of HK2 from mitochondria to cytoplasm during anoxia (Fig. 7C), and allows accurate quantification of the half-time for dissociation/reassociation. In contrast to HK2, HK1 is known to remain bound to mitochondria during metabolic stresses [3], [25] and does not translocate to the cytoplasm upon anoxia/reoxygenation. When HK1-YFP was overexpressed in AVRM and anoxia/reoxygenation was applied (Fig. 7A, upper series of images), the spatial variance measurement did not change (Fig. 7B and 7C).

**Figure 8:**
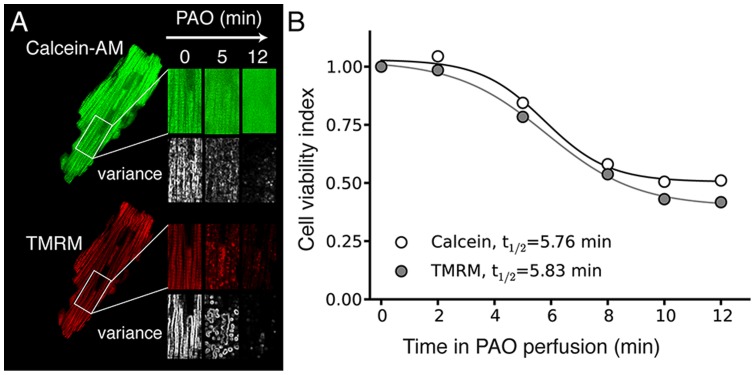
Calcein-based assessment of the mitochondrial permeability transition (MPT) in live adult cardiac myocytes. **A**) The mitochondrial network in AVRM was pre-loaded with Calcein-AM and then superfused with TMRM to record mitochondrial membrane potential. Upon exposure to 0.1 mM Phenylarsine Oxide (PAO) to induce MTP, calcein fluorescence redistributes within 10 min to the cytoplasm indicating MPT (upper series of images), and TMRM fluorescence concomitantly decreases dramatically indicating mitochondrial depolarization. Under each image, the corresponding spatial variance image is reported. **B**). Despite the differences in total fluorescence for the calcein and TMRM images, the corresponding spatial variance images predict similar times course of redistribution.

Finally, we also demonstrate that the spatial variance method works equally well for fluorescent probes which are loaded into mitochondria by other means than genetic encoding. Fig. 8 shows an example of an ARVM in which mitochondria were selectively loaded with the fluorescent dyes TMRM (30 nM) and calcein-AM. When exposed to Phenylarsine Oxide (PAO) to induce the mitochondria permeability transition (MTP), mitochondria depolarize causing loss of TMRM fluorescence from the mitochondrial matrix, with little change in cytoplasmic fluorescence (Fig. 8A, lower panel) (Due to its negative charge, TRMR is concentrated more than a thousand-fold in the matrix of polarized mitochondria, but when released is rapidly diluted). When calcein leaves the matrix, however, cytoplasmic fluorescence increases as mitochondrial fluorescence decreases (Fig. 8A, upper panel). Despite these differences in the pattern of fluorescence changes, both spatial variance measurements yielded comparable half-times of dissociation (5.76 min vs 5.83 min for the variance calculated from the calcein and TMRM data respectively), indicating the robustness of the technique (Fig. 8B).

## Discussion

Obtaining accurate quantitative data from live cell images is key for testing mechanistic hypotheses about molecular and cellular processes. For example, cell-based protein translocation assays can be used to probe cellular signaling pathways that are otherwise difficult to study using traditional biochemical assays. When tagged with fluorescent molecules, translocation of proteins of interest can be appreciated by visual inspection of images from fluorescence microscopy. However, these subjective impressions can be challenging to quantify. Here we developed a reliable and easy-to-implement image processing method to assess protein translocation between organelles and cytoplasm in living cells, using mitochondria as a test organellar system, based on the computation of the spatial variance of time-lapse images. We have demonstrated that the method is robust with respect to mitochondrial dynamics (fusion, fission, movement) and changes in cell morphology during the time-lapse imaging. We validated the method by quantifying HK2 dissociation and re-association from mitochondria during anoxia/reoxygenation as well as mitochondria transition pore opening in living cells, demonstrating its usefulness for both genetically-encoded and standard fluorescent probes.

The spatial variance method has the following advantages. (i) The method minimizes user bias by eliminating the need to choose and adjust ROI inside the cells as required by the ratiometric method. Indeed, spatial variance provides a global measure of spatial fluorescence heterogeneity which is relatively insensitive to changes in organellar and cellular morphology. (ii) The method can be applied to individual single cells. Single cell imaging techniques overcome the averaging effects inherent in ensemble measurements and enable characterization of the biological variability between individual cells. (iii) The method is capable of giving high detection responses in the setting of low contrast edges, as illustrated in detecting the mitochondrial network in low contrast regions of images. This is because edge detection is implemented on the basis of local variances rather than on the local gradients. As a result, ramp edges with low variation can be detected efficiently; [iv] The method is readily applied to the whole cell, providing a global measure of shifts in fluorescence compared to the ROI method or calculation of the variance along a single line drawn through the cell (see fig. 5 of [27]).

In their recent report, Venable and al. (2013) showed the pitfalls of using spatial averaged mean fluorescence imaging for detecting subcellular behavior of fluorescent probes, in particular because averaged spatial fluorescence cannot readily assess the degree of dye redistribution if the images are not resolved enough to detail all the compartments [28]. While our method is also based on the measure of a change in an average, using the variance instead of the raw fluorescence still permits detection of a change in compartment even if the total amount of fluorophore/dye remains unchanged (Figure 2B), as the variance images highlight the changes in distribution of the content in fluorescence between different compartments. In addition, the advantage of using the variance is that it can detect either a redistribution of the fluorophore from one compartment to another, or a loss of the fluorophore. This is demonstrated in the Figure 8, where the variance method performs equally well for both scenarios simultaneously, detecting the redistribution of Calcein and the loss of TMRM during the induction of PTP opening in adult cardiac myocytes.

In summary, the ease of implementation of this method should enable its application to a broad spectrum of time-lapse imaging studies. Although to date we have only validated the method for the mitochondrial network, we expect that it will prove valuable for measuring translocation between other organellar compartments as well.

## Supporting Information

Figure S1 **Implementation of a Gaussian window (weighted kernel) preserves edges better compared to a uniformly weighted windows (unity kernel), especially for large window sizes.** Window sizes from (3×3 pixels) to (29×29 pixels) were tested.
(TIF)

Figure S2 **Fluorescence bleaching correction.** **A**) Linear bleaching over time was simulated on our model by dividing the reference image with increasing factors (y = 0.25x+1). **B, D**) Without normalizing to the total fluorescence of the image, the intensity of the variance map images decreases over time (B), with the values reported in D. **C, E**) Normalizing to the total fluorescence of the image allows to correct for bleaching in the variance map images (C), as seen in the values (E).
(TIF)

Figure S3 **Comparison spatial variance and ratiometric ROI methods.** **A**) ROI chosen for the cytoplasmic (1) and mitochondrial (2) area. **B**) Detection of hexokinase (HK) dissociation with the variance (black) and ratiometric ROI (blue) image processing methods. **C**) The spatial variance method is insensitive to mitochondrial movement whereas the ratiometric ROI method is strongly distorted by mitochondrial movement.
(TIF)

Figure S4 **Pair-wise comparison of the variance mean computed with a Gaussian-weigthed (G) and a unity (U) kernel from images simulating translocation of a protein from fixed mitochondria to the cytosol.** **A**) The dashed red line is the line of equality on which all points would lie if the two kernels gave exactly the same variance mean for each image. As shown by data, the unity and Gaussian kernel give similar results in this scenario where the mitochondria are fixed. **B**) Graphical depiction of differences between paired observations from the two methods versus their average (left) and histogram of those differences (right). The mean of the differences (orange square and orange dashed line) is not statistically different from zero (red dashed line), revealing that there is no constant bias when using the unity kernel to calculate the variance compared to the Gaussian kernel. The slope of the regression of differences on means (gray line) is also non different from zero, indicating an absence of proportional biais. These results suggest that there is a very high degree of agreement between the unity and the Gaussian kernel variance computation when measuring the translocation of fluorophore/dyes from non motile compartments.
(TIF)

Figure S5 **Pair-wise comparison of the variance mean computed with a Gaussian-weigthed (G) and a unity (U) kernel from images simulating translocation of a protein from motile mitochondria to the cytosol.** **A**) The dashed red line is the line of equality on which all points would lie if the two kernels gave exactly the same variance mean for each image. As shown by data, the unity kernel tends to overestimate the variance compared to the Gaussian kernel especially for high values of variance. **B**) Graphical depiction of differences between paired observations from the two methods versus their average (left) and histogram of those differences (right). As shown by the slope of the regression line (gray) that differs significantly from zero (p<0.05), using a unity kernel instead of a gaussian-weighted kernel gives a proportional bias on the measure of the variance when compartments are motiles. In addition, the mean value for the differences (orange square and dashed line) also differs significantly from 0 (p<0.05), revealing a fixed (or ‘relative’) bias. Those biases might be the consequence of the excessive sensitivity of the unity kernel to image points near the edge of the unity window giving very high values when the simulated moving mitochondria suddenly enter the window.
(TIF)
