# Supplementary figures and images for: A Quantitative Method to Track Protein Translocation between Intracellular Compartments in Real-Time in Live Cells Using Weighted Local Variance Image Analysis
Source: PLoS One. 2013 Dec 20;8(12):e81988. doi: 10.1371/journal.pone.0081988 (PMC3869670; doi:10.1371/journal.pone.0081988)

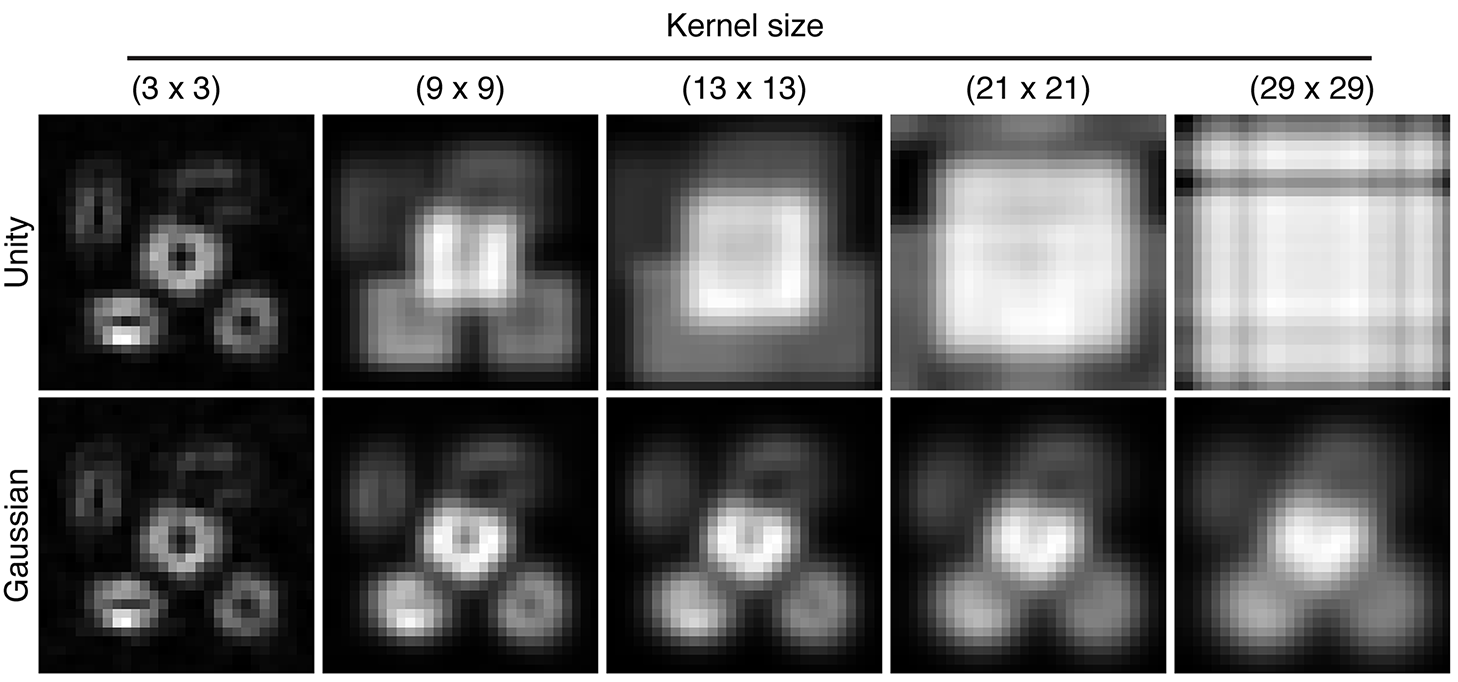

Supplement: Figure S1 — Implementation of a Gaussian window (weighted kernel) preserves edges better compared to a uniformly weighted windows (unity kernel), especially for large window sizes. Window sizes from (3×3 pixels) to (29×29 pixels) were tested. (TIF) [file pone.0081988.s001.tif]

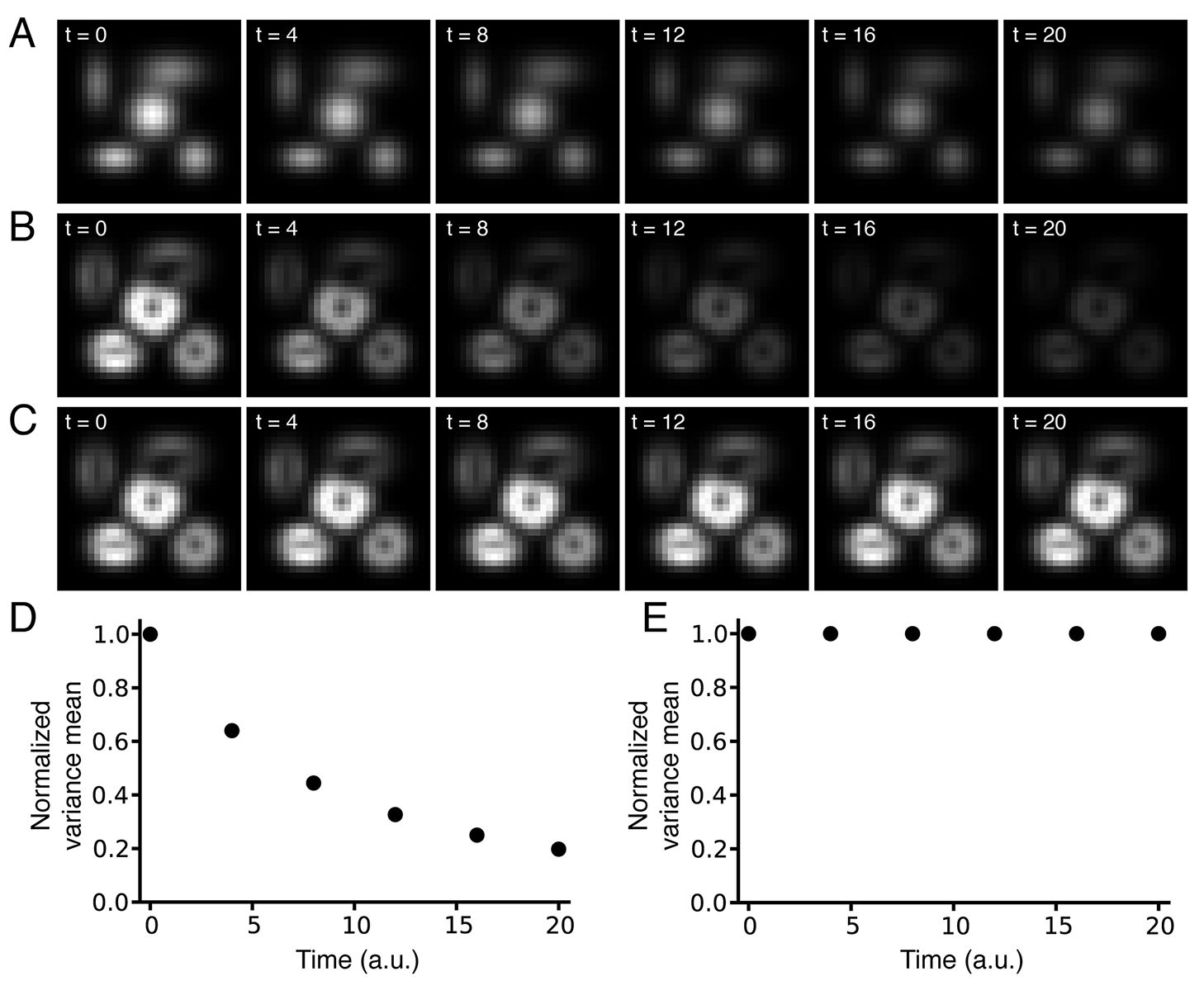

Supplement: Figure S2 — Fluorescence bleaching correction. A) Linear bleaching over time was simulated on our model by dividing the reference image with increasing factors (y = 0.25x+1). B, D) Without normalizing to the total fluorescence of the image, the intensity of the variance map images decreases over time (B), with the values reported in D. C, E) Normalizing to the total fluorescence of the image allows to correct for bleaching in the variance map images (C), as seen in the values (E). (TIF) [file pone.0081988.s002.tif]

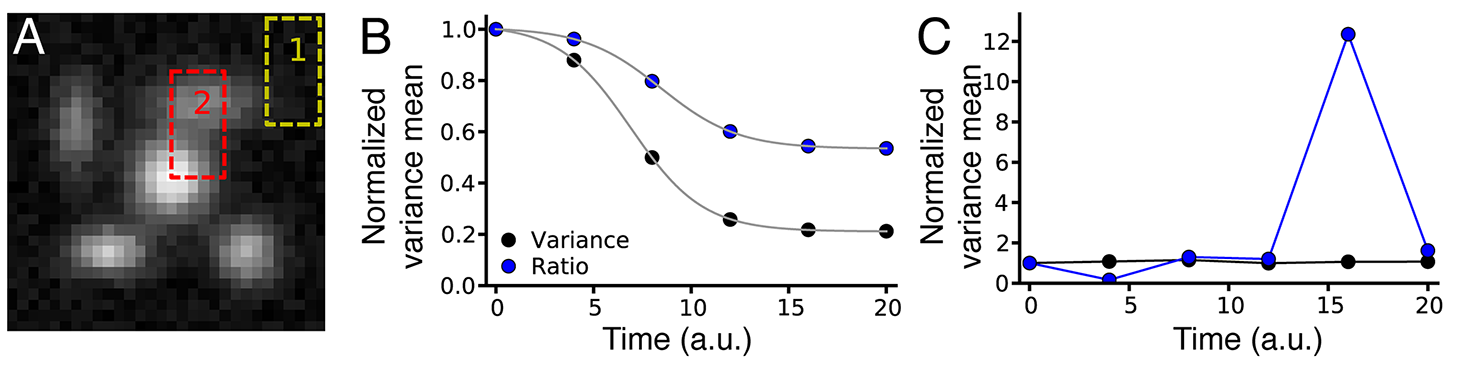

Supplement: Figure S3 — Comparison spatial variance and ratiometric ROI methods. A) ROI chosen for the cytoplasmic (1) and mitochondrial (2) area. B) Detection of hexokinase (HK) dissociation with the variance (black) and ratiometric ROI (blue) image processing methods. C) The spatial variance method is insensitive to mitochondrial movement whereas the ratiometric ROI method is strongly distorted by mitochondrial movement. (TIF) [file pone.0081988.s003.tif]

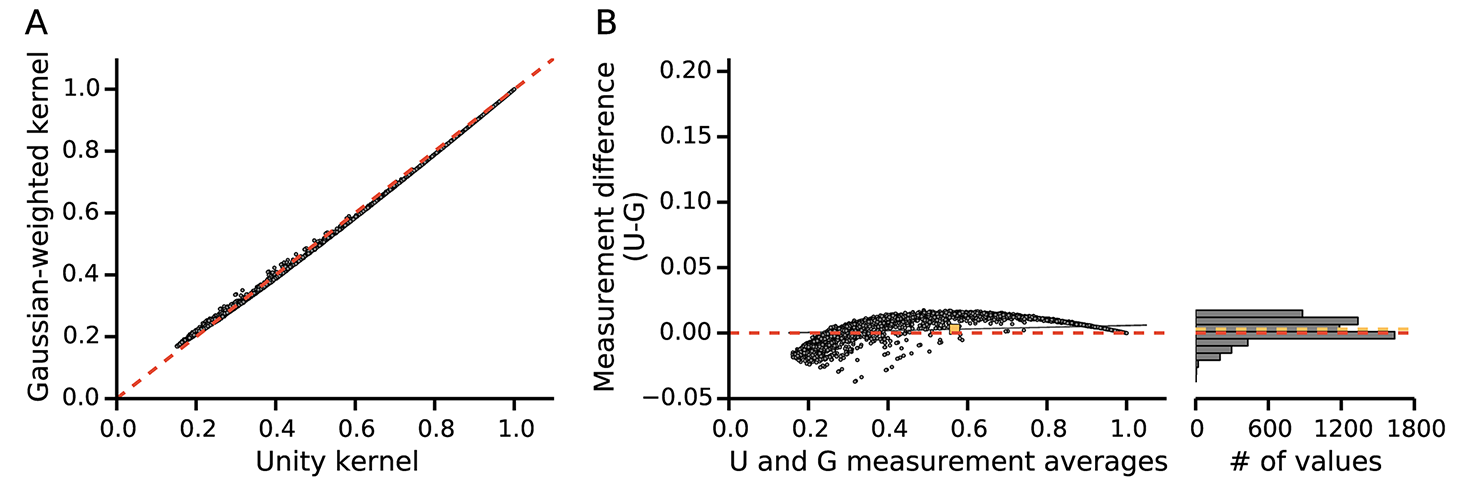

Supplement: Figure S4 — Pair-wise comparison of the variance mean computed with a Gaussian-weigthed (G) and a unity (U) kernel from images simulating translocation of a protein from fixed mitochondria to the cytosol. A) The dashed red line is the line of equality on which all points would lie if the two kernels gave exactly the same variance mean for each image. As shown by data, the unity and Gaussian kernel give similar results in this scenario where the mitochondria are fixed. B) Graphical depiction of differences between paired observations from the two methods versus their average (left) and histogram of those differences (right). The mean of the differences (orange square and orange dashed line) is not statistically different from zero (red dashed line), revealing that there is no constant bias when using the unity kernel to calculate the variance compared to the Gaussian kernel. The slope of the regression of differences on means (gray line) is also non different from zero, indicating an absence of proportional biais. These results suggest that there is a very high degree of agreement between the unity and the Gaussian kernel variance computation when measuring the translocation of fluorophore/dyes from non motile compartments. (TIF) [file pone.0081988.s004.tif]

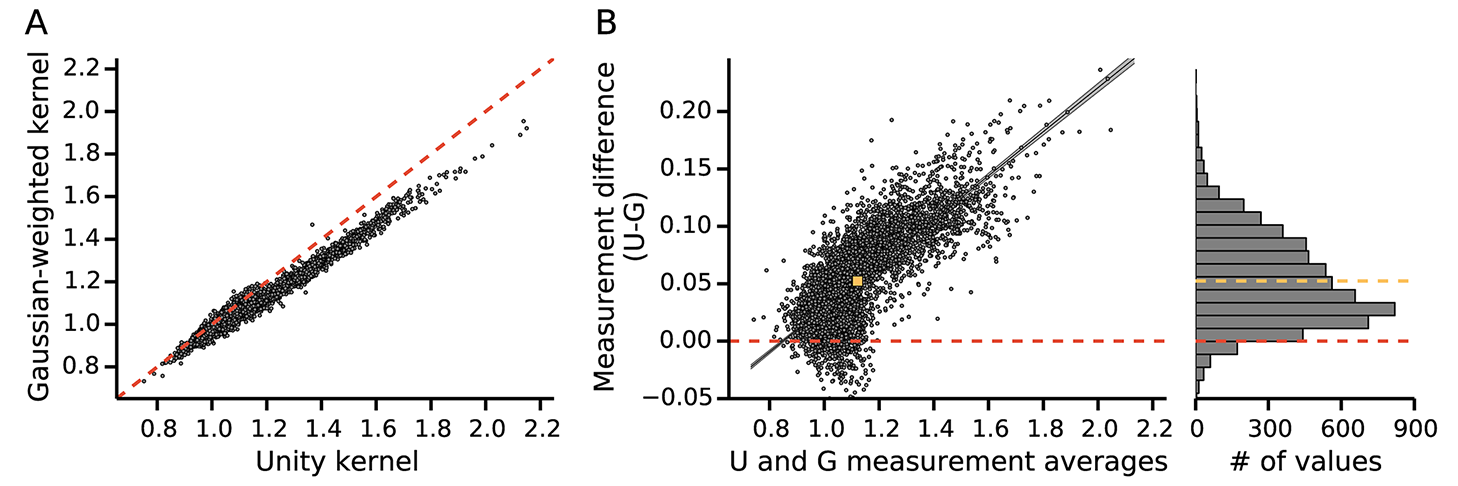

Supplement: Figure S5 — Pair-wise comparison of the variance mean computed with a Gaussian-weigthed (G) and a unity (U) kernel from images simulating translocation of a protein from motile mitochondria to the cytosol. A) The dashed red line is the line of equality on which all points would lie if the two kernels gave exactly the same variance mean for each image. As shown by data, the unity kernel tends to overestimate the variance compared to the Gaussian kernel especially for high values of variance. B) Graphical depiction of differences between paired observations from the two methods versus their average (left) and histogram of those differences (right). As shown by the slope of the regression line (gray) that differs significantly from zero (p<0.05), using a unity kernel instead of a gaussian-weighted kernel gives a proportional bias on the measure of the variance when compartments are motiles. In addition, the mean value for the differences (orange square and dashed line) also differs significantly from 0 (p<0.05), revealing a fixed (or ‘relative’) bias. Those biases might be the consequence of the excessive sensitivity of the unity kernel to image points near the edge of the unity window giving very high values when the simulated moving mitochondria suddenly enter the window. (TIF) [file pone.0081988.s005.tif]
